# Inhibitory effects of climate change on the growth and extracellular enzyme activities of a widespread Antarctic soil fungus

**DOI:** 10.1111/gcb.15456

**Published:** 2020-12-18

**Authors:** Marta Misiak, William P. Goodall‐Copestake, Tim H. Sparks, M. Roger Worland, Lynne Boddy, Naresh Magan, Peter Convey, David W. Hopkins, Kevin K. Newsham

**Affiliations:** ^1^ British Antarctic Survey NERC Cambridge UK; ^2^ Cardiff School of Biosciences Cardiff UK; ^3^ Institute of Zoology Poznań University of Life Sciences Poznań Poland; ^4^ Museum of Zoology University of Cambridge Cambridge UK; ^5^ Applied Mycology Group, Environment and AgriFood Theme Cranfield University Cranfield UK; ^6^ Scotland's Rural College Edinburgh UK; ^7^ Present address: Scottish Association for Marine Science Oban Argyll PA37 1QA UK

**Keywords:** Antarctica, climate warming, cold adaptation, extracellular enzymes, growth inhibition, psychrotrophy, soil fungi, water

## Abstract

Temperatures approaching or exceeding 20°C have been measured during summer in polar regions at the surfaces of barren fellfield soils under cloudless skies around solar noon. However, despite the upper temperature limit for the growth of cold‐adapted microbes—which are abundant in polar soils and have pivotal roles in nutrient cycling—typically being close to this temperature, previous studies have not addressed the consequences of climate change for the metabolism of these organisms in the natural environment. Here in a 5‐year field experiment on Alexander Island in the southern maritime Antarctic, we show that the abundance of *Pseudogymnoascus roseus*, the most widespread decomposer fungus in maritime Antarctic fellfield soils, is reduced by 1–2 orders of magnitude when irrigated and nutrient‐amended soils are warmed to >20°C during summer. Laboratory experiments under conditions mimicking those during midsummer in the natural environment indicated that the hyphal extension rates of *P. roseus* isolates and the activities of five extracellular enzymes are reduced by 54%–96% at high water availability after exposure to temperatures cycling daily from 2 to 21°C and 2 to 24°C, relative to temperatures cycling from 2 to 18°C. Given that the temperatures of surface soils at the study site already reach 19°C during midsummer, the observations reported here suggest that, at predicted rates of warming arising from moderate greenhouse gas emissions, inhibitory effects of climate change on the metabolism of *P. roseus* could manifest themselves within the next few decades. Furthermore, with peak temperatures at the surfaces of fellfield soils at other maritime Antarctic locations and in High Arctic and alpine regions already exceeding 20°C during summer, the observations suggest that climate warming has the potential to inhibit the growth of other cold‐adapted microbes, with negative effects on soils as the Earth's climate continues to warm.

## Graphical abstract

Summertime temperatures at the surfaces of barren maritime Antarctic soils can approach or exceed 20°C under cloudless skies around solar noon. Here we show that the metabolism of the cold‐adapted and widespread Antarctic soil fungus *Pseudogymnoascus roseus* is inhibited when it is exposed to temperatures cycling daily to more than 20°C, both in the natural environment and the laboratory, with substantial reductions in the in vitro growth rates of colonies and activities of extracellular enzymes. We propose that surface warming in cold regions has the potential to inhibit the growth of other cold‐adapted microbes, with negative effects on soils as the Earth's climate continues to warm.

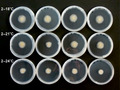

## 1 INTRODUCTION

During the latter half of the 20th century, maritime Antarctica was one of the fastest warming regions on Earth, with increases in near‐surface mean annual air temperatures of 1–3°C being recorded between the early 1950s and the turn of the millennium (Adams et al., 2009). Consistent with the current hiatus in warming at the global scale (Kosaka & Xie, 2013), this warming trend slowed in the late 1990s (Turner et al., 2016), but climate models forced with only moderate greenhouse gas emission scenarios predict further increases in air temperatures of 2–4°C in the region by the end of the 21st century (Bracegirdle et al., 2008; Bracegirdle & Stephenson, 2012). Rising air temperatures, associated either with long‐term warming trends (Adams et al., 2009) or short‐term heatwaves (King et al., 2017; Robinson et al., 2020), have already exerted substantial effects on the maritime Antarctic physical environment, with widespread glacial recession, ice shelf disintegration, increased snow melt, and more frequent precipitation having been recorded in the last 60 years (Adams et al., 2009; Cook et al., 2005; Fox & Cooper, 1998; Medley & Thomas, 2019; Thomas et al., 2008).

In contrast to its effects on the physical environment, the influence of climate change on maritime Antarctic terrestrial biota is much less clearly defined. It is known that higher plant populations have already expanded in response to warming and precipitation (Amesbury et al., 2017; Cannone et al., 2016) and that the photosynthetic rate of mosses increases in response to rising temperatures (up to 20–30°C; Perera‐Castro et al., 2020). Conversely, lichens appear to be sensitive to warming (Bokhorst et al., 2016; Colesie et al., 2018), and the size and activity of the soil microbial community either increases or decreases in response to warming, depending on water and nutrient availability (Benhua et al., 2014; Dennis et al., 2013; Yergeau et al., 2012). However, the influence of climate change on the metabolism of soil decomposer fungi in the maritime Antarctic natural environment has hitherto received no attention. The hyphae of these microbes, microscopic tubular cells that collectively attain lengths of up to 6.3 km g^−1^ dwt soil in maritime Antarctica (Dowding & Widden, 1974), ramify through soil and dead organic matter, secreting extracellular enzymes that break down organic compounds and release nutrients in forms that can be assimilated by plants and other soil microbes. They are thus pivotal to the functioning of all terrestrial ecosystems (Swift et al., 1979) and the current lack of information on how they will respond to climate change in maritime Antarctica represents a significant gap in knowledge.

A consistent feature of the fungi, and other microbes, inhabiting cold regions is their adaptation to growth at low temperatures. A rich literature, predominantly derived from the Arctic, has accumulated about the metabolism of cold‐adapted microbes at temperatures close to freezing point (e.g., Foster et al., 2016; McMahon et al., 2009; Mikan et al., 2002; Schimel et al., 2007). These organisms, referred to as psychrophiles and psychrotrophs, are able to grow at, or below, 0°C and typically have growth temperature optima at <15°C and <20°C, respectively (Morita, 1975; Russell, 1990). However, at temperatures above 20°C, the growth of psychrophiles halts entirely, and that of psychrotrophs, which are widespread in cold regions (Robinson, 2001), frequently declines (Kerry, 1990; Zucconi et al., 1996). As the arid fellfield soils that frequently form in polar regions lack plant cover, and are exposed during summer to higher total daily solar energy input than those in tropical or temperate zones, temperatures either approaching or exceeding 20°C can be reached at their surfaces around solar noon under cloudless skies during midsummer (Bokhorst et al., 2011, 2013; Convey et al., 2018). Given that climate change will force further increases in soil surface temperatures (Fang et al., 2019; Qian et al., 2011), it follows that the exposure of fellfield soils in polar regions to temperatures exceeding 20°C has the potential to influence the future growth and enzyme synthesis of psychrophilic and psychrotrophic microbes, with consequent effects on soil nutrient cycling. However, to the best of our knowledge, this possibility has never been tested in the natural environment.

Here, in a 5‐year field study in the southern maritime Antarctic, we test the hypothesis that temperatures exceeding 20°C will inhibit the growth and extracellular enzyme activities of the soil decomposer fungus *Pseudogymnoascus roseus* Raillo. Water, simulating increased snow melt and precipitation, and the substrates glucose, glycine, and tryptone soy broth (TSB), simulating increased organic carbon (C) and nitrogen (N) inputs from decomposing vegetation and algal cells (Hopkins et al., 2008), were applied to soil in factorial combination with warming to predict how *P. roseus* will respond to rising temperatures in a wetter and more biologically productive maritime Antarctic. Laboratory experiments, in which an emphasis was placed on mimicking the water potentials and diurnally cycling temperatures that are encountered in the natural environment during midsummer, were used to support the field observations. We focused on *P. roseus* because it is the most widespread maritime Antarctic fellfield soil decomposer fungus, with a DNA sequencing study showing it to be the only fungal taxon present in all 29 fellfield soils sampled from along a transect between Signy Island at 60°S and Alexander Island at 72°S (see figure S3 of Newsham et al., 2016), and because allied species in the *P. roseus* complex (notably *Pseudogymnoascus pannorum*; Minnis & Lindner, 2013), are psychrotrophs that have frequently been isolated from Antarctic, High Arctic, and alpine soils (e.g., Azmi & Seppelt, 1997; Domsch et al., 2007; Dowding & Widden, 1974; Edgington et al., 2014; Flanagan & Bunnell, 1980; Kerry, 1990; Kirtsideli, 2009; Zucconi et al., 1996).

## 2 METHODS

### 2.1 Site description

**FIGURE 1:**
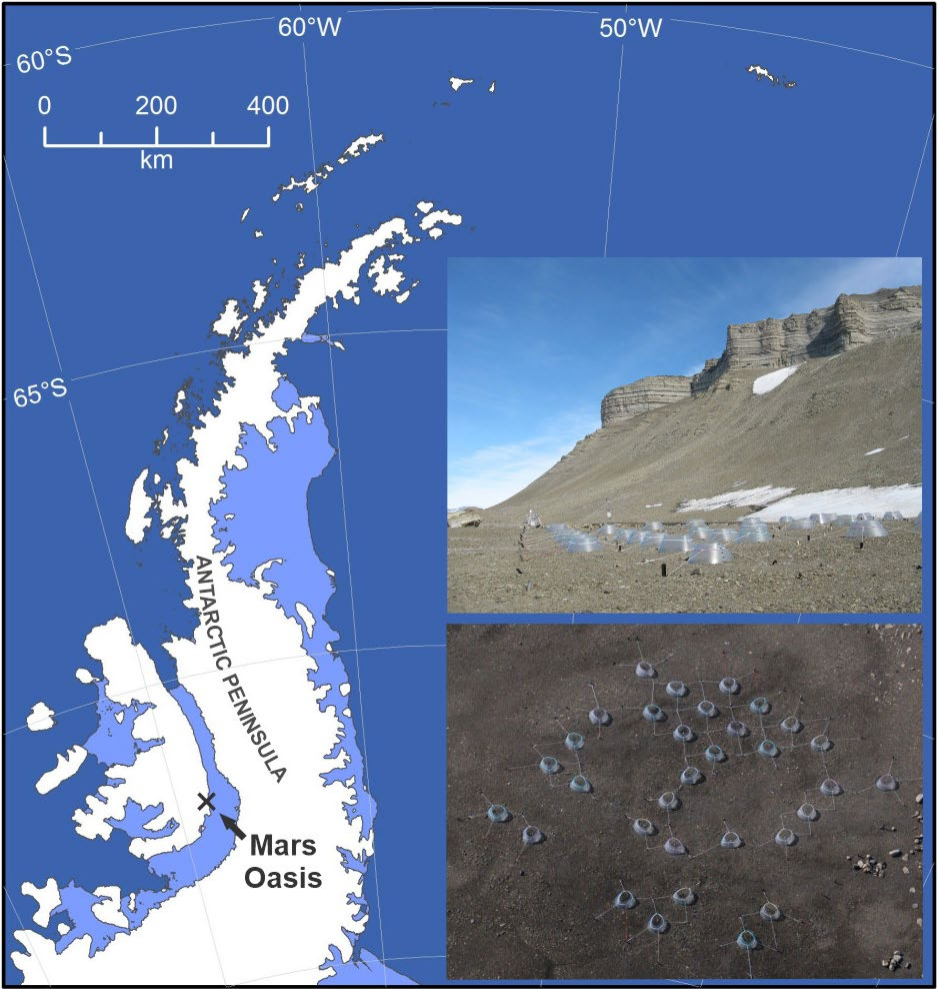
Map of the Antarctic Peninsula showing the position of Mars Oasis. Insets show ground‐level and aerial views of the experiment, which used 32 open top chambers to warm soil

Mars Oasis is located on the south‐eastern coast of Alexander Island in the southern maritime Antarctic at 71°52′42″S, 68°15′00″W (Figure 1). The oasis has a continental climate characterized by frequent periods of cloudless skies during summer. It consists of an upper and a lower terrace, with the upper terrace situated at the base of Two Step Cliffs and the lower terrace located adjacent to the grounding line between George VI Ice Shelf and Alexander Island (Sugden & Clapperton, 1981). The lower terrace consists of level areas of moraine, with coarse, angular debris, fluvio‐glacial till and lacustrine sediments, and ice‐cemented permafrost at 100–300 mm below the moraine surface (Sugden & Clapperton, 1981). Sparse stands of mosses are present close to a large pond on the lower terrace (Convey & Smith, 1997). Vertebrates, including seals and nesting birds, are absent from the oasis.

Mean annual air temperature at Mars Oasis is c. −10°C, with mean spring, summer, autumn, and winter air temperatures of c. −11°C, −1°C, −8°C, and −19°C, respectively (Convey et al., 2020; Hughes & Lawley, 2003). Annual precipitation, which is difficult to measure in Antarctica (Turner et al., 2002), has not been recorded at the oasis, but has been estimated at <200 mm water equivalent for Ablation Valley, 118 km to its north (Smith, 1988). The soil on the lower terrace at Mars Oasis has a mean pH of 7.9, an electrical conductivity of 31.9 μS, ${\text{NO}}_{3}^{‐}$‐N and ${\text{NH}}_{4}^{+}$‐N concentrations of 0.007 and 0.095 mg kg^−1^, and total organic C, N, phosphorus, and potassium concentrations of 0.26%, 0.02%, 8.01%, and 0.22%, respectively (Dennis et al., 2013; Newsham et al., 2010).

### 2.2 Field experiment

The experiment was established in late November 2007 on a level, homogeneous expanse of barren moraine soil on the lower terrace at Mars Oasis (Figure 1, insets), which enabled a high number of replicates of each treatment to be applied, reducing variation between replicate soil samples and allowing robust statistical analyses of the data derived from the experiment. The oasis was accessed by fixed‐wing, ski‐equipped aircraft deployed from Rothera Research Station on Adelaide Island. Sixty‐four circular plots (1 m diameter) were established in an area measuring 17 m × 17 m, 32 of which were covered with conical polycarbonate open top chambers (OTCs) of 1 m diameter (Figure 1, insets). In early December 2007, 2009, 2010, 2011, and 2012, shortly after snow melt and before substrates and water were applied, sterile plastic tubes (50 ml capacity) were filled with soil (depth 0–50 mm) from each plot and were capped tightly. Soil in each of 16 plots then either remained unamended or received glucose, glycine (the most frequent amino acid in maritime Antarctic soils; Hill et al., 2019), or powdered TSB (a mixture of amino acids and proteins) by mixing powder with sterile spoons into the soil to a depth of c. 30 mm. This process elevated soil organic C concentrations to 0.39% (glucose), 0.36% (glycine and TSB), and soil organic N concentrations to approximately 0.08% (glycine) and 0.05% (TSB). Unamended soil was also mixed to c. 30 mm depth with sterile spoons. Shortly after substrate application, soil moisture concentrations in half of the plots were raised to 100% of water holding capacity by applying 1.5 L of deionized water onto the soil surface in 0.05 m^2^ subplots. The experimental layout resulted in 16 OTC‐irrigation‐substrate treatment combinations, each replicated four times in a randomized design, including four unmanipulated plots. Soil temperatures were recorded by burying loggers (TinyTag Plus 2 TGP‐4017, Gemini Data Loggers Ltd.) in three chambered and three unchambered plots. The loggers, which were replaced with newly calibrated units each year, recorded temperatures hourly between December 2009 and December 2012. Their upper surface was located at 10–15 mm below the soil surface, with temperatures being recorded at a depth of 25–30 mm. The loggers did not become exposed at the soil surface. Data obtained in this way from December 2007 to December 2008 are reported by Dennis et al. (2013). The tubes containing the soil samples were kept at c. −3°C for 16–24 h (2007, 2009, 2011, and 2012) or 96 h (2010) before being returned to Rothera Research Station, where they were frozen at −20°C, prior to transport to the UK at the same temperature. They were subsequently defrosted, and water potential, an expression of biological water availability in which more negative values denote drier soils, was measured immediately using a dew point potentiometer (WP4C; Decagon Devices, Inc.).

Snow depth at Mars Oasis was determined from images of graduated stakes inserted into the ground (Figure S1) taken automatically once or twice daily between January 2003 and December 2008 using a digital camera (EI‐2000; Pentax). Both the camera and the window of the sealed housing in which it was located were heated, and flash illumination was used during winter. Soil freeze–thaw events were calculated from the number of times per week that hourly measured soil temperatures in unchambered and chambered plots fell below 0°C and then returned to temperatures above 0°C. The dates of soil thaw in late spring were estimated from when soil temperatures exceeded 0°C for four or more consecutive hours. Aerial images of the oasis taken during overflights confirmed that these dates coincided with the loss of snow cover from the soil surface.

### 2.3 Quantitative polymerase chain reaction assays

Quantitative (Q)‐polymerase chain reaction (PCR) assays were used to estimate the concentration of *Pseudogymnoascus* DNA present in soil. Novel Q‐PCR primers (forward: 5′‐GTCATTACAACCCTCAAGCTCAG‐3′; reverse: 5′‐GCGAGAAGAATTACTACGCTCG‐3′) were designed for this purpose using Geneious v8. DNA for the assays was extracted from defrosted soil (1.1 g fwt) collected in 2007 and 2009–2012 using a PowerSoil DNA isolation kit (MO BIO Laboratories Inc.). Q‐PCR amplification was performed in 48‐well plates using an Eco 48 machine (PCRMax Ltd.) and SYBR Green chemistry (Agilent Technologies). Reactions contained 0.5 µl of template DNA, 5 µl of SYBR Green master mix, 0.2 µl of each primer (final concentration 0.2 µM), and 4.1 µl of nuclease‐free water. The PCR amplification program consisted of denaturation at 95°C for 10 min followed by 35–40 cycles at 95°C for 0.5 min, annealing at 59°C for 0.5 min and elongation at 72°C for 0.5 min, followed by the generation of melting curves to verify amplification specificity. Each reaction was carried out in triplicate. A standard curve was produced using a dilution series of DNA (range of input DNA concentrations 3.5 × 10^−4^–35.0 ng) extracted from sterile soil from Mars Oasis spiked with *P. roseus* DNA extracted from colonies of the isolates (see Section 2.4). The software package Eco Study (PCRmax Ltd.) was used to generate quantitation cycle (Cq) values from the triplicate reactions, which were converted into estimates of DNA concentration, expressed as ng g^−1^ dwt soil, from the standard curve.

### 2.4 Isolation and identification of *P. roseus*

Three *P. roseus* isolates, referred to below as isolates 1–3, were obtained from Mars Oasis soil using the soil plate method (Warcup, 1950). Defrosted soil collected in 2009 from three glycine‐ and three TSB‐amended plots was sieved (2 mm) and, using a microspatula, c. 4.5 mg (fwt) was added to the surface of Czapek–Dox agar medium to which Rose Bengal had been added (1:15,000) in 90 mm non‐vented Petri dishes. The soil was spread evenly on the agar medium surface and the dishes were incubated upside down at 7°C. After 16 days, three morphologically similar colonies from two glycine‐amended plots and one TSB‐amended plot were isolated onto half strength potato dextrose agar (PDA) medium and kept at c. 12°C. DNA was extracted from c. 400 mg fwt hyphae scraped from the surfaces of colonies growing on sterile cellophane overlaying half strength PDA medium using a DNeasy plant mini kit (Qiagen). Fungal DNA between the 18S and 28S rRNA genes, including the internal transcribed spacer (ITS) regions 1 and 2, was PCR‐amplified from the DNA extracts using a MyTaqMix (Bioline) kit following the manufacturer's protocol and with the ITS1F (5′‐CTTGGTCATTTAGAGGAAGTAA‐3′)/ITS4 (5′‐TCCTCCGCTTATTGATATGC‐3′) primer pair (Gardes & Bruns, 1993; White et al., 1990). The thermocycling program consisted of an initial denaturation of 94°C for 5 min, then 35 cycles of denaturation at 94°C for 1 min, annealing at 54°C for 1 min, and elongation at 72°C for 3 min, followed by a final elongation at 68°C for 10 min. Negative controls, which consisted of sterile water in place of template DNA, did not yield amplicons. The amplicons were bidirectionally sequenced at a commercial facility.

Following manual inspection and editing, consensus sequences derived from the bidirectional sequencing reads, which have been deposited in GenBank (accessions MT477869, MT477886, and MT477911), were analyzed in UNITE (https://unite.ut.ee/), a curated database of fungal ITS sequences. These analyses showed 99% identities with *Pseudogymnoascus* sequences from soils, air, and marine sponges in maritime and continental Antarctica, Glacier National Park in Canada, and Svalbard in the High Arctic, with the closest UNITE species hypotheses for all three isolates being *P. roseus* (SH1557165.08FU). Microscopic examination of the conidia of isolates 1–3 indicated that they are smooth walled, globose to ellipsoidal, measure 3.0–4.5 × 2.0–2.5 µm, and have truncate bases, all defining features of *P. roseus* (Domsch et al., 2007). Note that *P. pannorum* (syn. *Geomyces pannorum*), which has been widely reported from Antarctica (e.g., Azmi & Seppelt, 1997; Kerry, 1990; Zucconi et al., 1996), is considered to be a member of the *P. roseus* species complex (Minnis & Lindner, 2013).

### 2.5 Hyphal extension rate analyses

The *P. roseus* isolates were grown on soil extract medium (SEM) and exposed to temperatures cycling daily from 2–18°C, 2–21°C, and 2–24°C in factorial combination with four water potential treatments (−1.06, −3.60, −6.00, and −10.80 MPa). These treatments were chosen to reflect the temperatures and water potentials measured in Mars Oasis soil during midsummer. To obtain inoculated plugs of medium without dry spores on their surfaces, sporulating colonies of isolates 1–3 growing on half strength PDA medium were flooded with sterile water and the colony surfaces were rubbed with a sterile spreader, prior to spreading 200 µl of the spore suspension onto half strength PDA medium, which was incubated at c. 8°C for 16 h. Circular plugs (5 mm diameter) of each isolate were then bored from the medium and were placed onto the centers of sterile cellophane film (55 mm × 55 mm, Natureflex 28 NP; Innovia Films) overlaying sterile black cloth and capillary matting (Gardman) in 90 mm diameter non‐vented Petri dishes. The cloth and matting had previously had 14 ml of SEM applied to them. The SEM had been prepared by adding 500 g (fwt) of soil from Mars Oasis to 1 L of spring water, autoclaving the suspension for 20 min at 121°C, standing for at least 24 h and then passing the supernatant through a 0.4 μm filter. Prior to re‐autoclaving, sucrose (0.1 g) and yeast extract (0.01 g) were added to 100 ml of the filtrate, along with KH_2_PO_4_ (0.02 g) and polyethylene glycol 8000 (38.6, 54.1, 68.7, or 98.0 g, adjusting water potentials to −1.06, −3.60, −6.00, and −10.80 MPa, respectively). Total organic C and N contents per dish were 59 and 2.1 mg, respectively, compared with organic C and N contents in the same volume of glycine and TSB‐amended soils at Mars Oasis of 69 and 6.6 mg, respectively. The dishes were sealed with Parafilm and incubated in the dark in temperature‐controlled cabinets (MCR‐350 Versatile Environmental Test Chamber; Sanyo) for up to 10 weeks. Cabinet temperatures, which were monitored every 10 min using TinyTag Plus 2 TGP‐4017 loggers, were programmed to cycle daily from 2–18°C, 2–21°C, or 2–24°C, with dwells at 2°C, 18°C, 21°C, and 24°C of 6 h duration. Colonies were photographed weekly using a digital camera (SX700 HS; Canon), with image analysis (ImageJ; National Institute of Health) subsequently being used to measure colony areas. Areas were regressed against time (all *r* ^2^ ≥ 0.95) to calculate increases in hyphal extension rates (expressed as mm^2^ day^−1^).

As a corollary to these analyses, isolates 1–3 were also grown on SEM in 0.2 µm vented sterile cell culture flasks (25 cm^2^ area, 130189; Thermo Scientific BioLite) at constant temperatures of −2°C, 2°C, 4°C, 9°C, 12°C, 15°C, 18°C, 21°C, and 24°C. The flasks at −2°C, 2°C, 9°C, and 12°C were submerged vertically into coolant in recirculating water baths, with flask necks protruding from the coolant. Temperatures of the SEM were monitored regularly using a thermometer set into SEM in a dummy flask. Flasks at other temperatures were incubated in temperature‐controlled cabinets, the temperatures of which were monitored hourly with TinyTag Plus 2 TGP‐4017 loggers. Colony diameters were measured weekly up to 42 days, making two measurements across each colony at a 90° angle to each other, using a dissecting microscope to visualize the colony front. Colony areas were subsequently estimated, accounting for the area of the 5 mm diameter plugs, and hyphal extension rate (in mm^2^ day^−1^) was calculated. The water potential of the medium, which was unadjusted with polyethylene glycol 8000, was −0.14 MPa.

### 2.6 Extracellular enzyme analyses

At the end of the experiments described in Section 2.5 in which *P. roseus* was exposed to fluctuating temperatures, extracellular enzymes were extracted from hyphae by placing the cellophane on which the isolates were growing upside down in 4 ml of 10 mM phosphate buffer (pH 7.2), followed by shaking in the dark at 7°C for 2 h. The extracts were stored at −80°C prior to analysis. Following previously described methods (Alam et al., 2009), the activities of β‐1,4‐glucosidase (cellulase), *N*‐acetyl‐β‐glucosaminidase (chitinase), leucine aminopeptidase, and alkaline and acid phosphatases were measured using *p*‐nitrophenyl substrates. Extracellular enzyme activities were measured in 96 well microplates, using three technical replicates. Enzyme extract (40 μl) was mixed with each enzyme substrate (40 μl) and made up to 100 μl with 10 mM sodium acetate buffer (pH 6 for acid phosphatase or pH 8 for other enzymes) and incubated, along with appropriate controls, for 15–120 min at 37°C. The reactions were stopped by adding 10 μl of 1 M sodium bicarbonate solution to each well and absorbance readings were recorded immediately using a microplate reader (Sunrise; TECAN) at a wavelength of 405 nm. The activity of each enzyme was calculated from *p*‐nitrophenol standard calibration curves (ranging from 0 to 1.5 μmol ml^−1^ of *p*‐nitrophenol) and expressed as μmol of *p*‐nitrophenol released h^−1^. These data were then expressed as specific enzyme activities, which account for the amount of protein present in the extract. Protein content was measured using a Micro BCA Protein Assay Kit (ThermoFisher) in triplicate by adding 100 μl of working solution (BCA reagents A, B and C, mixed in a 25:24:1 ratio, respectively) to 100 μl of extract in a microplate. Microplates with samples and controls were incubated for 120 min at 37°C and absorbance measured at 562 nm. Protein concentration was calculated from an albumin calibration curve (range 0–200 μg ml^−1^). Specific enzyme activity was expressed as units of each enzyme [U] released h^−1^ mg^−1^ protein.

### 2.7 Conidial production and germination experiments

To determine the effects of the temperature cycle and water potential treatments (see Section 2.5) on the numbers of *P. roseus* conidia produced per colony, 20 µl of the phosphate buffer that was used to extract enzymes from colonies (see Section 2.6) was added to a chamber of a disposable hemocytometer (Kova International) and the number of conidia present in an area of 2 mm^2^ was counted using phase contrast microscopy at ×400 magnification. The number of conidia produced per colony was subsequently calculated. To determine treatment effects on conidial germination, aliquots (200 μl) of SEM modified to water potentials of −1.06, −3.6, −6.00 and −10.80 MPa were inoculated with conidia (1–2 × 10^6^ ml^−1^) in sterile 2 ml capacity Eppendorf tubes. At each water potential, 15 tubes per isolate were inoculated, with five of these 15 tubes each subsequently being incubated at the 2–18°C, 2–21°C, and 2–24°C temperature cycles. Germination was measured daily between 2 and 6 days (at −1.06 MPa), at 4, 7, 10, and 14 days (at −3.60 MPa), at 10, 13, 17, 20, and 23 days (at −6.00 MPa), and at 7, 10, 14 days, and weekly thereafter up to 42 days (at −10.80 MPa). At each sampling, 20 µl of SEM from each tube was added to a chamber of a disposable hemocytometer and 50–164 conidia were scored for germination at ×400 magnification under phase contrast, with germinated conidia being defined as those having germ tubes exceeding the width of the conidium. A total of 62,315 conidia were scored for germination.

### 2.8 Statistical analyses

Data from Q‐PCR analyses were not normally distributed, and hence the main and interaction effects of OTCs, irrigation, and substrates on the concentrations of *P. roseus* DNA in soil were analyzed using factorial contrasts in ANOVA based on bootstrapping procedures of 10,000 randomizations of log(*x* + 1)‐transformed data. The main and interaction effects of temperature cycles and water potentials on hyphal extension rates and specific enzyme activities were analyzed separately for each isolate using general linear models on log(*x* + 1)‐transformed data, with Tukey's test being used to determine post hoc differences between means. Data for isolates 1–3 were then combined and one‐tailed *t*‐tests were used to determine differences between zero and the mean percentage changes in hyphal extension rates and specific enzyme activities between the 2–21°C and 2–18°C temperature cycles, and between the 2–24°C and 2–18°C temperature cycles. General linear models were used to test the main effects of temperature cycles and water potentials on log(*x* + 1)‐transformed conidial germination and production data, and the main effects of OTCs and irrigation on soil water potential. Repeated measures ANOVA, with plot nested in OTC, time and plot as random factors, and OTC as a fixed factor, was used to analyze the effects of OTCs on mean weekly soil temperatures and freeze–thaw events during the 29 weeks in December and January 2009–2012. Analyses were made in SPSS 25 (IBM) and MINITAB 18.

## 3 RESULTS

### 3.1 Soil temperatures, water potentials, and snow depth

**FIGURE 2:**
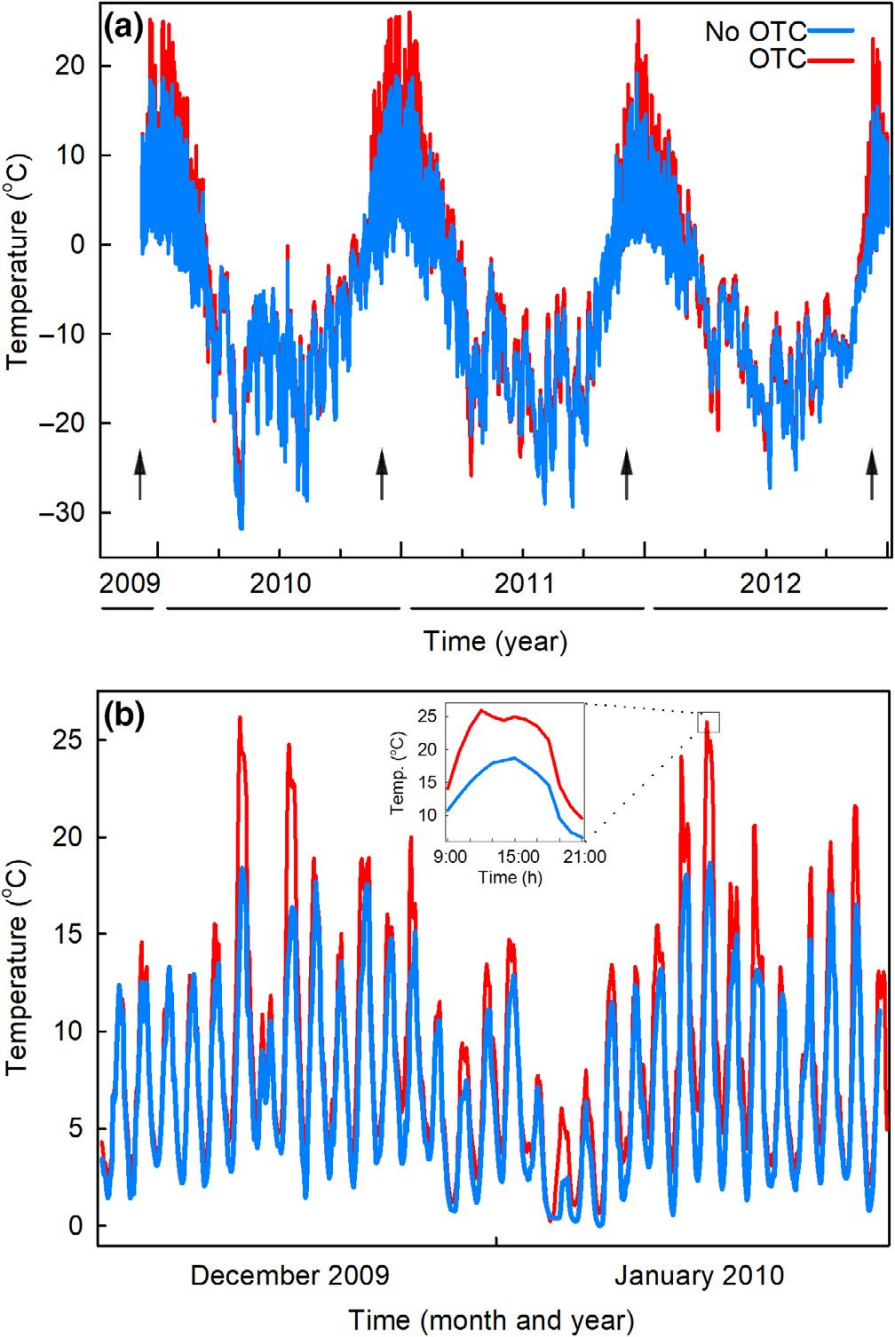
Soil temperatures measured at Mars Oasis in unchambered control plots and open top chamber (OTC) plots. (a) Soil temperatures recorded hourly at 25–30 mm depth between December 2009 and December 2012. Arrows denote samplings. (b) Soil temperatures recorded hourly at 25–30 mm depth between 15 December 2009 and 15 January 2010. Values shown in (a) and (b) are means of temperatures in three OTC plots and three unchambered plots. Inset in (b) shows mean soil temperatures recorded hourly from 9:00 to 21:00 on 8 January 2010

Mean annual surface soil temperatures measured in chambered and unchambered plots were −6.3°C and −7.3°C, with minimum temperatures in these plots being −28.3°C and −31.8°C, respectively. There were minimal effects of OTCs on soil temperatures between February and November, with OTCs principally affecting temperatures in December and January when ground was free of snow and ice (Figure 2a,b). Significantly higher weekly mean soil temperatures were recorded during these months in chambered plots compared with unchambered plots (7.3°C and 5.6°C, respectively, repeated measures ANOVA; *F*_1,140_ = 8.96, *p* = 0.040). However, these mean values masked considerable variation, with surface soil temperatures in OTCs frequently rising to >20°C during these months (Figure 2a,b). During December and January, surface soil temperatures of ≥20°C were recorded in OTCs for up to 38 h per month, with temperatures remaining at above 20°C for up to eight consecutive hours around solar noon under cloudless skies (Figure 2b, inset). The absolute maximum surface soil temperatures measured in chambered and unchambered plots over the duration of the experiment were 27.5°C and 19.2°C, respectively (Figure 2a,b).

Freeze–thaw events were infrequent in December and January (Figure 2a,b), with soil temperatures remaining continuously above freezing point for up to 7 and 4 weeks in chambered and unchambered plots, respectively (Figure S2a). When soil was not permanently frozen during these months, OTCs reduced the number of freeze–thaw events by a third, from a mean of 2.7 events to 1.8 events per week (repeated measures ANOVA; *F*_1,185_ = 22.87, *p* = 0.009). Significant effects of OTCs on freeze–thaw events were recorded in 8 weeks between December 2009 and December 2012, when soil temperatures fell to between −0.7°C and −4.7°C in OTCs and control plots (Figure S2a,b). In late 2010, soil thaw was advanced by 9 days in chambered plots compared with unchambered plots, with full thaw occurring in these plots on 22 November and 1 December, respectively (*F*_1,5_ = 67.6, *p* = 0.001). No differences in the timing of soil thaw were recorded in other years (all *F*_1,5_ < 5.5 and *p* > 0.08; data not shown).

**FIGURE 3:**
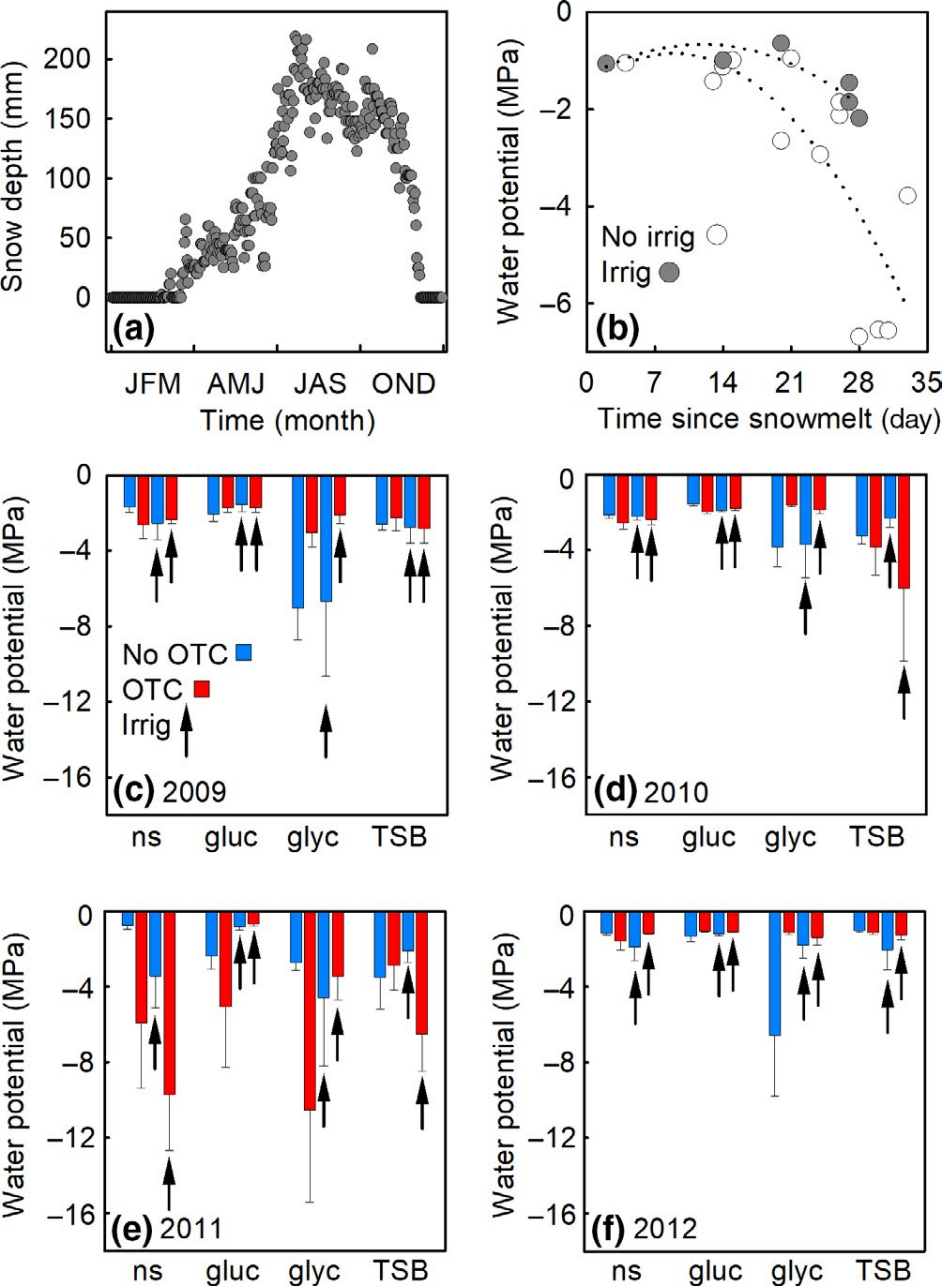
Snow cover and soil water potentials measured at Mars Oasis. (a) Mean daily snow depth at Mars Oasis measured over 6 years (2003–2008). (b) Water potentials measured in irrigated and unirrigated soils as a function of time since snow melt. Dotted lines are second order polynomial fits. (c–f) Water potentials measured at the samplings in 2009–2012 in soils receiving no substrate (ns), glucose (gluc), glycine (glyc), or tryptone soy broth (TSB) and a factorial combination of warming (applied with open top chamber [OTCs]) and irrigation (irrig). Values are means of four replicates ± SEM

In accordance with the surface soil temperature data, analyses of images of Mars Oasis confirmed that snow cover was routinely absent from ground between early December and mid‐February (Figure 3a). During this period, in 6 years of monitoring, sparse snow accumulated on ground on 4 days (with full melt within 24 h on each occasion), and one light rainfall event was recorded. Snowfall events became frequent from March, with snow accumulating to a mean depth of 220 mm during July (Figure 3a). Snow melt started in October, with rapid melt occurring during November (Figure 3a). Water was lost rapidly from unirrigated soils in the 5 weeks following snow melt, with mean soil water potential declining from −1 to <−4 MPa during this period (Figure 3b). In contrast, mean water potentials in irrigated soils remained between −1 and −2 MPa for up to a month following snow melt (Figure 3b). Measurements of soil water potentials prior to irrigation at each sampling ranged between −0.7 and −10.4 MPa (mean −3.0 MPa), with no detectable effects of previous irrigation on this variable (Figure 3c–f). Two‐way ANOVA indicated marginally significant effects of OTCs on water potentials in glycine‐amended soils at the samplings in 2009 and 2010, when OTCs increased soil water potential from −6.8 to −2.6 and −3.8 to −1.7 MPa, respectively (*F*_1,12_ = 3.78 and 4.03, *p* = 0.076 and 0.068, respectively; Figure 3c,d).

### 3.2 Q‐PCR analyses

**FIGURE 4:**
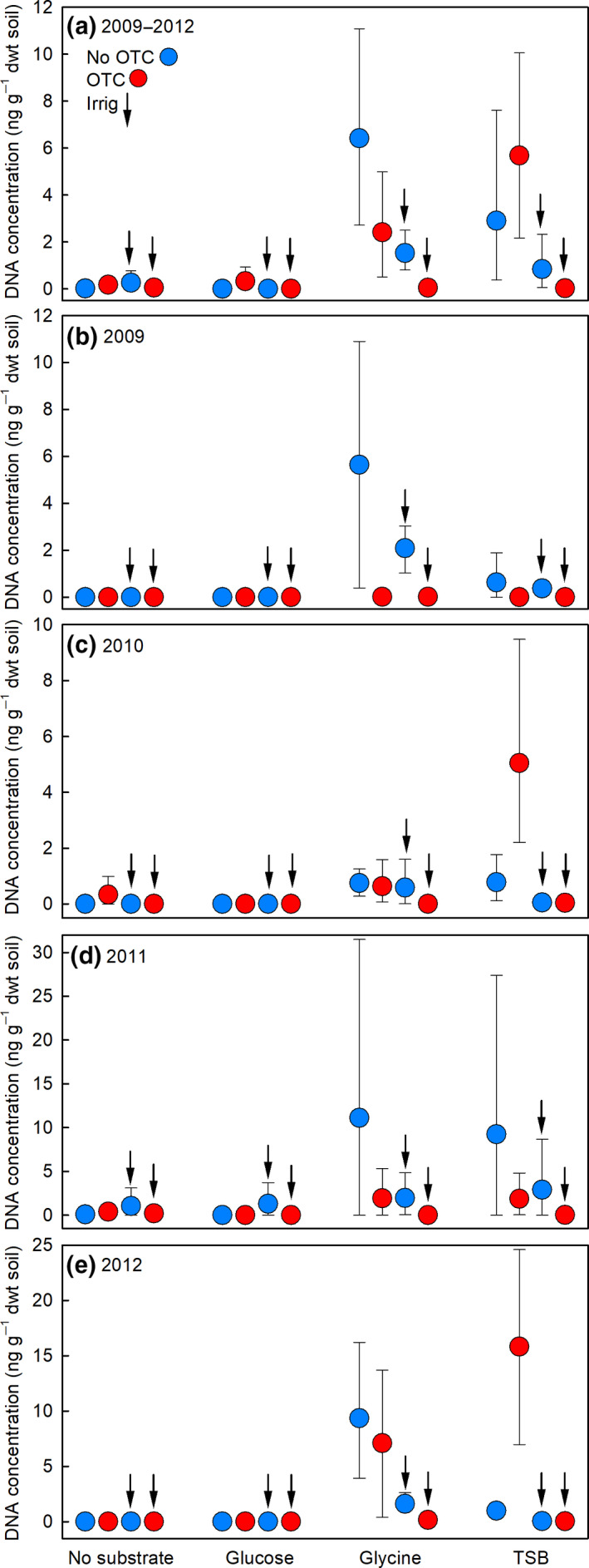
Concentrations of *Pseudogymnoascus roseus* DNA in Mars Oasis soil measured using quantitative polymerase chain reaction assays. Values are DNA concentrations (a) averaged across 2009–2012 and in (b–e) individual years in soils receiving no substrate, glucose, glycine, or tryptone soy broth (TSB) and a factorial combination of warming (applied with open top chambers, OTCs) and irrigation (irrig). Values are means of four replicates ± 95% bootstrap confidence intervals. See Table 1 for an ANOVA table summarizing the treatment effects

Quantitative‐polymerase chain reaction analyses showed that the application of organic C in the form of glucose only affected the concentration of *P. roseus* DNA in soil at the final sampling of the experiment in 2012, when glucose application caused a significant 0.26‐fold reduction in DNA concentration (Table 1). No interaction effects of glucose application with the OTC and irrigation treatments were recorded (Table 1). In contrast, the application of organic C and N to soil in the form of glycine led to marked and highly significant increases in the concentration of *P. roseus* DNA in soil, with main effects of the substrate being recorded across all samplings and in individual years except 2011 (Table 1; Figure 4a–e). Increases in DNA concentration in response to glycine application ranged between 5.9‐fold and 1.8 × 10^4^‐fold (Table 1). However, significant glycine × OTC interaction terms recorded across all samplings and in 2009 and 2012 (Table 1) indicated that OTCs caused significant reductions in the concentration of *P. roseus* DNA in glycine‐amended soils (Figure 4a,b,e). Furthermore, the significant glycine × irrigation interaction terms recorded across all samplings and in 2012 (Table 1) showed that irrigation caused reductions in *P. roseus* DNA concentrations in glycine‐amended soil (Figure 4a,e). Similar effects were recorded in TSB‐amended soil: main effects of TSB were found across all samplings and in each year except 2011, with increases in the concentrations of *P. roseus* DNA between 1.8 × 10^1^‐fold and 1.1 × 10^4^‐fold in response to the substrate (Table 1; Figure 4a–e). The significant TSB × OTC interaction term recorded in 2009 (Table 1) indicated that OTCs caused a reduction in the concentration of *P. roseus* DNA in TSB‐amended soil (Figure 4b), and the significant TSB × irrigation interaction terms across all samplings and in 2012 (Table 1) showed that irrigation caused reductions in DNA concentrations in TSB‐amended soils (Figure 4a,e).

**TABLE 1:** Significance of main and interaction effects of three substrates, open top chambers (OTCs), and irrigation on the concentrations of *Pseudogymnoascus roseus* DNA in soil at Mars Oasis averaged across all samplings (2009–2012) or in individual years. Values in bold are significant at *p* < 0.05. Note that the directions of changes in DNA concentrations in response to the main effects of the treatments and the two‐way interactions are shown as arrows (↑, increase; ↓, decrease), with fold changes in response to the main effects being shown after the arrows

	2009–2012	2009	2010	2011	2012
Glucose	0.522	0.199	0.661	0.406	**0.005** (↓, 2.6 × 10^−1^)
Glucose × OTC	0.234	0.451	0.322	0.436	0.261
Glucose × irrigation	0.333	0.462	0.529	0.414	0.875
Glucose × OTC × irrigation	0.779	0.547	0.193	0.557	0.729
Glycine	**<0.001** (↑, 2.1 × 10^1^)	**<0.001** (↑, 1.8 × 10^4^)	**0.002** (↑, 5.9)	0.102	**<0.001** (↑, 1.3 × 10^4^)
Glycine × OTC	**<0.001** (↓)	**0.001** (↓)	0.100	0.083	**0.043** (↓)
Glycine × irrigation	**0.011** (↓)	0.629	0.122	0.231	**0.036** (↓)
Glycine × OTC × irrigation	0.392	0.403	0.459	0.766	0.456
TSB	**<0.001** (↑, 1.9 × 10^1^)	**0.005** (↑, 2.4 × 10^3^)	**<0.001** (↑, 1.8 × 10^1^)	0.244	**<0.001** (↑, 1.1 × 10^4^)
TSB × OTC	0.393	**0.027** (↓)	0.671	0.555	0.175
TSB × irrigation	**0.026** (↓)	0.203	0.067	0.184	**<0.001** (↓)
TSB × OTC × irrigation	0.296	0.482	0.636	0.680	**0.020**

Only one three‐way interaction was recorded between the substrates, OTCs, and irrigation on *P. roseus* DNA concentrations, with a significant TSB × OTC × irrigation interaction term being recorded in 2012 (Table 1). Despite this, across all years, the additive effect of OTCs and irrigation in glycine‐ and TSB‐amended soils reduced DNA concentrations by 99.3%–99.6%, relative to soils receiving these substrates that had not been warmed or irrigated (Figure 4a). Strikingly, over the course of the experiment, the mean concentrations of *P. roseus* DNA in chambered, irrigated soils that had been amended with glycine and TSB were consistently 1–2 orders of magnitude lower than in unchambered, unirrigated soils that had received these substrates (Figure 4b–e). Over the duration of the experiment, the absolute maximum concentration of *P. roseus* DNA measured in chambered, irrigated, and glycine‐ or TSB‐amended soils never exceeded 0.3 ng g^−1^ dwt soil, compared with absolute maximum DNA concentrations in unchambered and unirrigated soils receiving glycine or TSB of 31.5 and 36.4 ng g^−1^ dwt soil, respectively.

### 3.3 Hyphal extension rates

**FIGURE 5:**
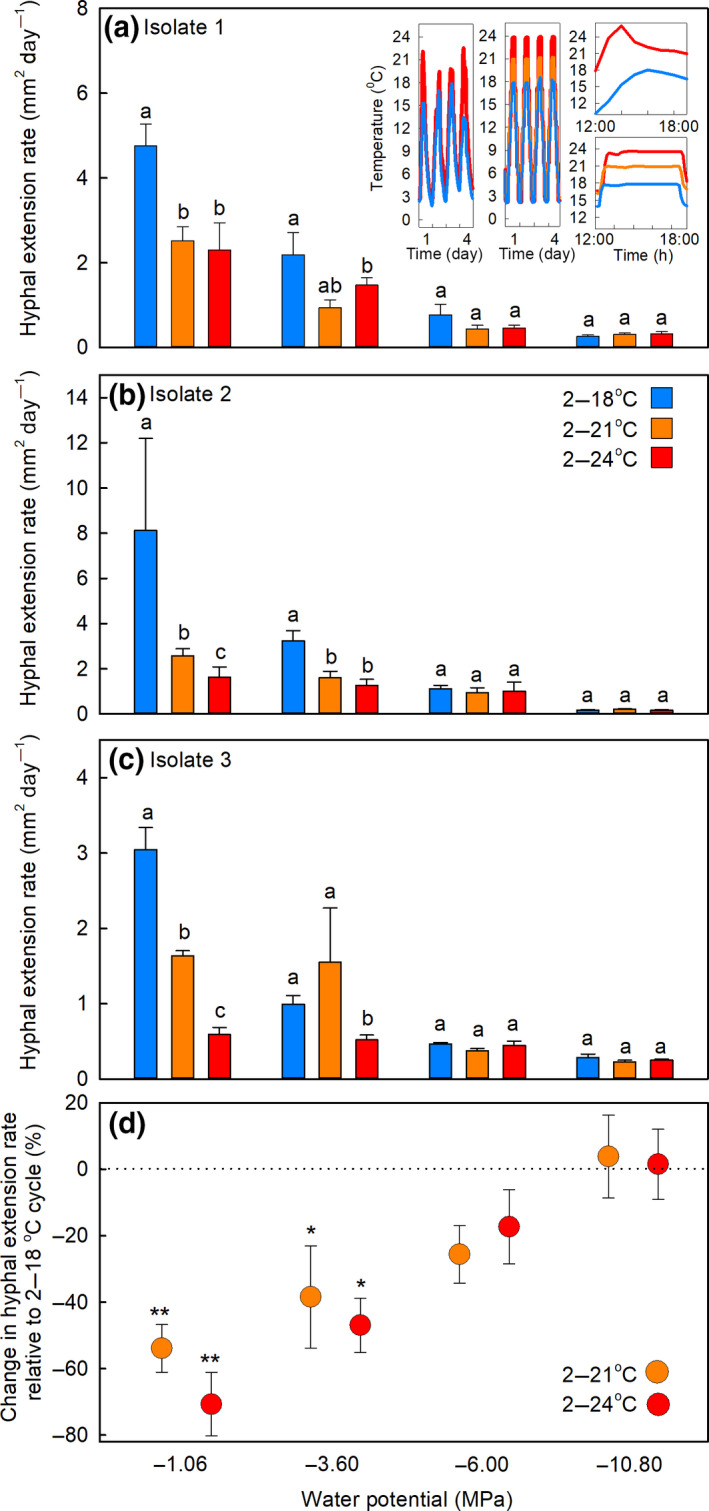
Hyphal extension rates of *Pseudogymnoascus roseus* isolates under controlled conditions. (a–c) Mean extension rates of *P. roseus* isolates 1–3 grown on soil extract medium and exposed to daily temperature cycles of 2–18°C, 2–21°C, or 2–24°C at water potentials of −1.06, −3.60, −6.00, or −10.80 MPa. Values are means of three replicates ± SEM, with distinct letters in (a)–(c) denoting significant (*p* < 0.05) differences within each group of three values. The insets in (a) show the mean temperatures recorded in three open top chamber (OTC) and three unchambered control plots at Mars Oasis from 20 to 23 January 2010 (left), the temperatures recorded hourly over 4 days in controlled environment cabinets cycling daily from 2–18°C, 2–21°C, or 2–24°C (middle), the mean temperatures in three OTC plots and three unchambered control plots on 13 January 2011 (top right) and the temperatures recorded over 6 hours in controlled environment cabinets (bottom right). (d) Mean percentage changes in hyphal extension rates across the three isolates exposed to daily temperature cycles of 2–21°C and 2–24°C, relative to the 2–18°C temperature cycle. Asterisks indicate significant differences from zero, denoted by the dotted horizontal line, tested with one‐tailed *t*‐tests (*p < 0.05 and **p < 0.01)

When water was freely available, in vitro exposure to temperatures cycling daily to >20°C, which were similar in amplitude to the temperatures measured in soils at Mars Oasis during midsummer (Figure 5a, insets), strongly inhibited the hyphal extension rates of isolates 1–3 (Figure 5a–d). Highly significant effects of temperature cycles (all *F*_2,35–36_ > 7.84, *p* < 0.002) and water potentials (all *F*_3,35–36_ > 30.52, *p* < 0.001) were recorded on the hyphal extension rates of all three isolates. The temperature cycle × water potential interaction also influenced the extension rates of isolates 1 and 3 (both *F*_6,35–36_ > 2.71, *p* < 0.028; Figure 5a,c) and marginally affected that of isolate 2 (*F*_6,35_ = 2.30, *p* = 0.056; Figure 5b). At −1.06 MPa, exposure to both the 2–21°C and 2–24°C cycles consistently reduced the hyphal extension rates of all three isolates compared with the 2–18°C temperature cycle, and, at −3.60 MPa, exposure to the 2–24°C cycle reduced the extension rates of the isolates, relative to the 2–18°C cycle (Figure 5a–c). No effects of the 2–21°C and 2–24°C cycles were recorded on hyphal extension rates, compared with the 2–18°C temperature cycle, at −6.00 and −10.80 MPa (Figure 5a–c). Analyses of data for all three isolates indicated that exposure to the 2–21°C and 2–24°C cycles caused growth inhibitions, expressed as mean percentage reductions in hyphal extension rate relative to the 2–18°C cycle, of 54%–71% at −1.06 MPa and 38%–47% at −3.60 MPa (Figure 5d). In contrast, at lower water availabilities (−6.00 and −10.80 MPa), exposure to the 2–21°C and 2–24°C cycles did not inhibit hyphal extension rate, relative to the 2–18°C cycle (Figure 5d). Experiments in which isolates 1–3 were exposed to constant temperatures between −2°C and 24°C for up to 6 weeks indicated negligible but measurable growth at −2°C, maximum growth rate at 18°C, and sharp declines in growth rate at >21°C (Figure S3).

### 3.4 Extracellular enzymes

Isolates 1–3 strongly synthesized cellulase, chitinase, and leucine aminopeptidase, with, at the 2–18°C temperature cycle, maximum specific activities recorded for these enzymes of 1170, 480, and 225 U h^−1^ mg^−1^ protein, respectively (Figure S4). Phosphatase activities were lower, with maximum specific activities of 45 and 18 U h^−1^ mg^−1^ protein for alkaline and acid phosphatases, respectively, at 2–18°C (Figure S4). With the exception of cellulase and acid phosphatase activities for isolate 3, temperature cycles had a significant effect on specific enzyme activities (all *F*_2,39–42_ = 3.89–33.67, *p* < 0.029; Figure S4). Water potential similarly had significant effects on the activities of all five extracellular enzymes (all *F*_3,39–42_ = 3.36–33.67, *p* < 0.028), often with increased activities of cellulase, chitinase, and leucine aminopeptidase as water potentials declined from −1.06 to −6.00 MPa (Figure S4). Except for chitinase and acid phosphatase activities for isolate 2, significant temperature cycle × water potential interactions were consistently recorded on specific enzyme activities (all *F*_6,33–36_ = 2.53–25.44, *p* < 0.039). These interactions were in part owing to reductions at −1.06 MPa in specific enzyme activities at the 2–21°C and 2–24°C temperature cycles, relative to the 2–18°C cycle, but, at −3.60 and −6.00 MPa, increased activities at the 2–21°C temperature cycle, relative to the 2–18°C and 2–24°C temperature cycles (Figure S4).

**FIGURE 6:**
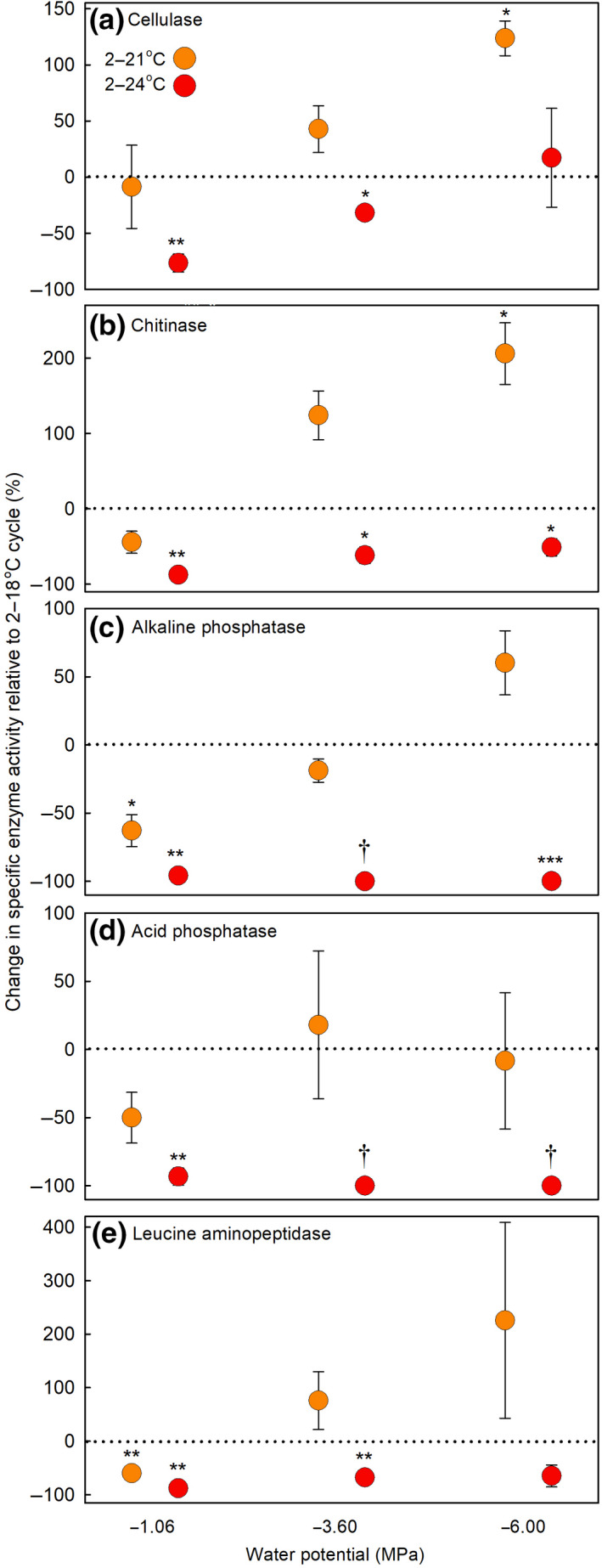
Changes in the specific activities of (a) cellulase, (b) chitinase, (c) alkaline phosphatase, (d) acid phosphatase, and (e) leucine aminopeptidase secreted by *Pseudogymnoascus roseus* under controlled conditions. Data shown are mean percentage changes in the specific activities of each enzyme extracted from hyphae exposed to daily temperature cycles of 2–21°C and 2–24°C, relative to the 2–18°C temperature cycle, at water potentials of −1.06, −3.60, and −6.00 MPa on soil extract medium. Values are means derived from the three isolates ± SEM, with asterisks indicating significant differences from zero, denoted by the dotted horizontal line, tested with one‐tailed *t*‐tests (*p < 0.05; **p < 0.01, and ***p < 0.001). Tests on values marked with an obelisk (†) were not possible owing to the absence of enzyme activity at the 2–24°C temperature cycle. Note that data are not shown for −10.80 MPa as there was little or no enzyme activity for either of the phosphatases or leucine aminopeptidase at this water potential. The specific enzyme activity data, from which the values shown here were derived, are shown in Figure S4

As for hyphal extension rates, the activities of the five enzymes were most strongly inhibited by temperatures cycling daily to >20°C at the highest water availability. Across all three isolates, at −1.06 MPa, exposure to the 2–24°C cycle caused consistent 76%–96% reductions in the activities of all five enzymes, compared with the 2–18°C cycle (Figure 6a–e). Also at −1.06 MPa, alkaline phosphatase and leucine aminopeptidase activities were reduced by 60%–63% by exposure to the 2–21°C cycle, relative to the 2–18°C cycle (Figure 6c,e). At moderate water availability (−3.6 MPa), exposure to the 2–24°C cycle, compared with the 2–18°C cycle, caused 32%–68% reductions in the activities of cellulase, chitinase, and leucine aminopeptidase (Figure 6a,b,e), and eliminated the activities of alkaline and acid phosphatases (Figure 6c,d; Figure S4). At −6.00 MPa, exposure to the 2–24°C cycle caused 52% and 99% reductions in the activities of chitinase and alkaline phosphatase (Figure 6b,c), and eliminated that of acid phosphatase, compared with the 2–18°C cycle (Figure 6d; Figure S4). However, exposure to the 2–21°C cycle at this water potential led to one‐ and twofold increases in the activities of cellulase and chitinase, relative to the 2–18°C cycle, respectively (Figure 6a,b).

### 3.5 Conidial production and germination

Conidial germination was influenced by both the temperature cycle and water potential treatments (Figure S5). The 2–21°C and 2–24°C temperature cycles increased germination rate relative to the 2–18°C temperature cycle (all *F*_2,288_ > 4.46, *p* < 0.012), and more negative water potentials caused reductions in germination rate (all *F*_2,288_ > 16.50, *p* < 0.001; Figure S5). The number of conidia produced per colony was either unaffected by the temperature cycle treatments for isolates 1 and 3 (both *F*_2,27_ < 1.18, *p* > 0.322; Figure S6), or, for isolate 2, was positively affected by increasing temperatures, with two‐ to threefold increases in the number of conidia at the 2–24°C cycle relative to the 2–18°C cycle for this isolate (*F*_2,25_ = 15.12, *p* < 0.001; Figure S6). Conidial production was lower at more negative water potentials, with 55%–88% reductions in the number of conidia produced per colony as water potential declined from −1.06 to −6.00 MPa (all *F*_2,27_ = 6.46, *p* < 0.005; Figure 6). Conidia were not produced at −10.80 MPa.

## 4 DISCUSSION

The observations from the field and laboratory experiments reported here collectively indicate that exposure to temperatures exceeding 20°C inhibits the growth and extracellular enzyme activities of *P. roseus*, the most widespread soil decomposer fungus in maritime Antarctic fellfield soils (Newsham et al., 2016). Antarctic and Arctic isolates of fungi in the *P. roseus* species complex, including *P. pannorum*, which typically have cosmopolitan distributions (see table S1 of Cox et al., 2019), have been widely reported to exhibit reduced growth when exposed in vitro to constantly applied temperatures above 20°C on artificial media under saturating nutrient conditions and at uncontrolled water potentials (Azmi & Seppelt, 1997; Bergero et al., 1999; Dowding & Widden, 1974; Edgington et al., 2014; Flanagan & Bunnell, 1980; Kerry, 1990; Kirtsideli, 2009; Zucconi et al., 1996). However, the data from the field experiment described here are the first in situ observations indicating inhibitory effects of exposure to temperatures exceeding 20°C on a psychrotrophic soil microbe in the natural environment. The field observations were corroborated by laboratory studies, in which isolates of *P. roseus* were grown at water potentials and diurnally fluctuating temperatures similar to those occurring in soils in the natural environment, both of which have significant effects on fungal growth (Burgess & Griffin, 1968; Manzoni et al., 2012).

Although these observations broadly confirmed our hypothesis of inhibited metabolism of *P. roseus* at temperatures exceeding 20°C, it is important to note that direct effects of warming on the fungus were not recorded, either in the laboratory or field experiments. In the former setting, the growth of the fungus was only suppressed by temperatures cycling daily to >20°C when water was freely available, and, in the field, only in irrigated soils to which organic C and N had been applied. Together, these observations broadly suggest that further warming of maritime Antarctica, in combination with organic C and N inputs from expanding plant populations (Amesbury et al., 2017; Cannone et al., 2016; Horrocks et al., 2020) and enhanced water availability arising from increased precipitation, snow melt or localized flooding (Adams et al., 2009; Fox & Cooper, 1998; Medley & Thomas, 2019; Robinson et al., 2020), is likely to inhibit the growth of *P. roseus* in soil. Given that soil surface temperatures at Mars Oasis already reach 19°C during summer, the observations suggest that surface warming of only a few degrees Celsius, equivalent to that anticipated in the maritime Antarctic over the next few decades under moderate greenhouse gas emission scenarios (Bracegirdle et al., 2008; Bracegirdle & Stephenson, 2012), will elicit these effects at the study site. However, superimposed over this long‐term rise in temperature will be the influence of heatwaves, such as those recorded in austral summer 2019/20, when air temperature close to the northernmost tip of the Antarctic Peninsula exceeded 20°C (Robinson et al., 2020).

Arctic field experiments have previously identified the importance of water availability in determining microbial responses to warming (Christiansen et al., 2017; Geml et al., 2015). In the field experiment reported here, in late spring 2011, when mean water potential across all plots was −4.1 MPa and, in chambered, unirrigated, and glycine‐amended soil, was −10.4 MPa—which approached the point at which microbial growth in mineral soils halts owing to diffusion limitation of solutes (Manzoni et al., 2012)—and no treatment effects were found on the concentration of *P. roseus* DNA in soil. In agreement with the laboratory study, the effects of OTCs and their interactions with organic C and N amendments and irrigation were restricted to summers 2009 and 2012, when mean soil water potentials across all plots were −2.8 and −0.7 MPa, respectively. In common with talus soils at high altitudes (Freeman et al., 2009; Ley et al., 2004), water was lost rapidly from unirrigated soils at Mars Oasis in the 5 weeks following snow melt in early December, with soil water potentials declining to between −4 and −6 MPa during this period. With summertime inputs of water in the form of snow and rain to Mars Oasis being very infrequent (Convey & Smith, 1997), it follows that the hyphal growth of *P. roseus* in unirrigated surface soils would have been restricted during this season. In contrast, water was lost less rapidly from irrigated soils, with soil water potential remaining at between −1 and −2 MPa during the month following snow melt, apparently allowing the inhibitory effects of warming to >20°C to manifest themselves on the hyphal growth of the fungus in chambered, irrigated soils. At present the mechanism responsible for the retention of water in previously irrigated soils is not clear, but, owing to the flat terrain of the lower terrace at Mars Oasis, it is possible that water applied to soil, instead of flowing downslope in subsurface water tracks (Levy et al., 2011), may have become trapped above the permafrost table at a depth of c. 100–300 mm (Sugden & Clapperton, 1981).

Although microbes are active in soils below freezing point (McMahon et al., 2009; Mikan et al., 2002; Schimel et al., 2007), the experiments at constant temperatures reported here showed that the hyphal growth of *P. roseus* is negligible at −2°C, with maximum hyphal extension rate occurring at 18°C. It is hence reasonable to assume that the treatment effects of OTCs on the concentration of *P. roseus* DNA in soil arose during summertime at Mars Oasis, when the fungus would have been most active and when the chambers exerted significant effects on soil temperatures. Thus, despite soils being continuously thawed for only 4–7 weeks each year during midsummer, we anticipate that the inhibitory effects of OTCs on the fungus in the field experiment were caused by increases in soil temperatures to >20°C for up to 38 h each month in hydrated, nutrient‐amended soils in summer, with laboratory experiments under conditions similar to those encountered in the field supporting this view. Nevertheless, OTCs do not solely affect soil temperatures, with other effects, such as delayed springtime soil thaw, occurring in chambers across multiple studies (Bokhorst et al., 2011, 2013). Here, we found that soil thaw was advanced, not delayed, in OTCs during one austral late spring. Marginally significant effects of OTCs were also recorded on water potentials in glycine‐amended soils at two samplings, with 2.1–4.2 MPa increases in water potentials in chambered soils to which glycine had been applied compared with unchambered soils receiving the amino acid, which would have amplified the deleterious effects of warming on *P. roseus* in these soils.

As observed previously at Mars Oasis (Bokhorst et al., 2011; Dennis et al., 2013), OTCs also significantly reduced the number of freeze–thaw events in chambered soil during late spring and late summer. Freeze–thaw is a significant factor for polar and montane soils, with the process reducing microbial biomass and leading to increased soil C and N availability following spring thaw (Grogan et al., 2004; Schimel et al., 2007). However, the extent of freeze–thaw effects depends on the severity of the freezing treatment and on the environment in which the experiments take place, with less pronounced effects in permanently cold soils (Matzner & Borken, 2008). It is possible that the 33% reduction in the number of freeze–thaw events in OTCs compared with control plots at Mars Oasis might have led to lower microbial mineralization of organic C and N, limiting nutrient availability in chambered, glycine‐, or TSB‐amended soils, and resulting in restricted growth of *P. roseus*. However, experiments on High Arctic tundra and montane soils indicate that freeze–thaw events with minimum temperatures of −9.0°C and −7.5°C have no effects on the microbial mineralization of C from glucose and amino acids, including glycine (Foster et al., 2016; Lipson & Monson, 1998). Given these observations, it seems unlikely that freeze–thaw events at Mars Oasis, during which soil temperatures fell to between −0.7°C and −4.7°C, would have affected the mineralization of nutrients from glycine and TSB in OTCs, with consequent impacts on the growth of *P. roseus* in soil.

The hyphae of decomposer fungi obtain nutrients and energy by ramifying through the soil matrix and foraging for organic matter. The analyses here indicate that exposure to temperatures above 20°C at high water availability will not only compromise the ability of *P. roseus* hyphae to forage for resources in maritime Antarctic soils by slowing hyphal extension rate, but will also inhibit the capacity of the fungus to decompose soil organic compounds, including phosphate group‐containing molecules, leucine residues in peptides and proteins, and cellulose and chitin, the two most ubiquitous natural polymers on Earth (Rinaudo, 2006). At high water availability, the specific activities of extracellular enzymes, including cellulase and chitinase, which are synthesized by Antarctic isolates of *P. roseus* and *P. pannorum* (Duncan et al., 2008; Newsham et al., 2018), were suppressed by temperatures cycling daily to 24°C. Those of phosphatases, which are also synthesized by other strains of Antarctic *P. pannorum* (Fenice et al., 1997), were entirely eliminated by the 2–24°C temperature cycle at moderate water availability. In contrast, and in agreement with studies showing that reduced water availability or reduced precipitation increases chitinase and cellulase activities (Alam et al., 2009; Ren et al., 2017), the combination of temperatures cycling daily to 21°C at a water potential of −6 MPa enhanced the specific activities of cellulase and chitinase, suggesting that rising temperatures in drier soils will initially stimulate the breakdown of cellulose and chitin by *P. roseus*.

The majority of fungal biomass in the soils of cold regions is considered to be present in a vegetative state, with, for example, up to 6.3 and 1.6 km hyphae g^−1^ dwt of soil at Signy Island in maritime Antarctica and Point Barrow in the High Arctic, respectively (Dowding & Widden, 1974). However, fungal conidia, reproductive cells that act as survival structures during winter in polar soils and have roles in long‐range dispersal (Bergero et al., 1999; Marshall, 1996), will usually also be present in these soils (Robinson, 2001). Although laboratory experiments showed clear effects of temperatures cycling daily to above 20°C on hyphal extension rates, they could not eliminate the possibility that the changes to the concentrations of *P. roseus* DNA observed in the field experiment might have arisen partly from changes to the frequencies or germination of conidia in soil. However, in agreement with previous experiments on Antarctic *Geomyces* isolates (Edgington et al., 2014), temperatures of >20°C stimulated the germination of *P. roseus* conidia, and, for one of the three isolates, increased the numbers of conidia produced per colony of fungus. Given these observations, it is reasonable to conclude that the effects of rising temperatures on *P. roseus* arose from inhibited hyphal growth of the fungus in soil.

An important caveat to the present study is that not all Antarctic soils are exposed to regular episodes of temperatures approaching 20°C during summer, as reported here for fellfield soils on Alexander Island. For instance, soils that support mosses and lichens, which typically have higher moisture concentrations than those studied here, are buffered by the shading effect of vegetation and the high heat capacity of water (Guglielmin et al., 2012; Perera‐Castro et al., 2020), with temperatures during summer at a depth of 2 cm in wet vegetated soils reaching 8.5–16.7°C, compared with 17.2–21.5°C in arid, bare soils at Edmonson Point in continental Antarctica (Cannone & Guglielmin, 2009; Hrbáček et al., 2020). Nevertheless, it is clear from the literature that the surfaces of maritime Antarctic fellfield soils with low albedos are exposed during summer to temperatures approaching and exceeding 20°C. For example, peak summertime temperatures of 17.4–24.6°C and 21.2–27.4°C have already been recorded in surface soils on Signy Island in the South Orkney Islands and the Léonie Islands in Marguerite Bay, respectively, with the temperatures of fellfield soils on southern Alexander Island reaching 21.8–24.3°C during summer (Bokhorst et al., 2011, 2013; Convey et al., 2018), and with temperatures at the surfaces of moss cushions on King George Island and Ardley Island reaching 20.4–34.2°C during summertime (Perera‐Castro et al., 2020). Depending on the duration of exposure to such temperatures, we anticipate inhibitory effects of further rises in surface temperatures in the region on the growth and activity of *P. roseus* and other psychrotrophic fungi, such as *Thelebolus microsporus*, *Cladosporium cladosporioides* and *C. herbarum* (Azmi & Seppelt, 1997; Kerry, 1990; Zucconi et al., 1996).

The findings here add to the steadily accumulating body of evidence that the upper thermal limits of the Earth's biota, such as tropical crops, coral reefs, lichens, and mid‐ and high‐latitude vegetation, will be exceeded as the planet warms (Bita & Gerats, 2013; Colesie et al., 2018; Hughes et al., 2003; O'Sullivan et al., 2017; Way & Sage, 2008). We propose that inhibitory effects of warming on cold‐adapted soil microbes are an insidious, hitherto unrecognized consequence of climate change for Antarctic terrestrial ecosystems, and that they may have a wider influence on soil ecology in other cold regions. Given peak summertime temperatures at soil surfaces in alpine regions and the Arctic far exceeding 20°C (e.g., 42°C on Svalbard, 47°C at Audkuluheidi on Iceland and 58°C in the Bogong High Plains of Australia; see figure S6 in Bokhorst et al., 2013), and the abundance of psychrotrophic microbes in the soils of these regions (Flanagan & Bunnell, 1980; Kirtsideli, 2009; Russell, 1990), we advocate further research to determine if inhibitory effects of warming on microbial metabolism could constrain the development of these soils and the terrestrial ecosystems that depend on them.

## Supporting information

Fig S1‐S6

## Data Availability

The data associated with this study are archived in the UK Polar Data Centre (https://doi.org/10.5285/1CF37889‐9F77‐41B9‐8C49‐B175FBD03406).
