# Supplementary material for: Inhibitory effects of climate change on the growth and extracellular enzyme activities of a widespread Antarctic soil fungus
Source: Glob Chang Biol. 2020 Dec 18;27(5):1111–25. doi: 10.1111/gcb.15456 (PMC7898924; doi:10.1111/gcb.15456)

Supplementary Figures S1–S6 for Misiak et al., *inhibitory effects of climate change on the growth and extracellular enzyme activities of a widespread Antarctic soil fungus*

**

**

**
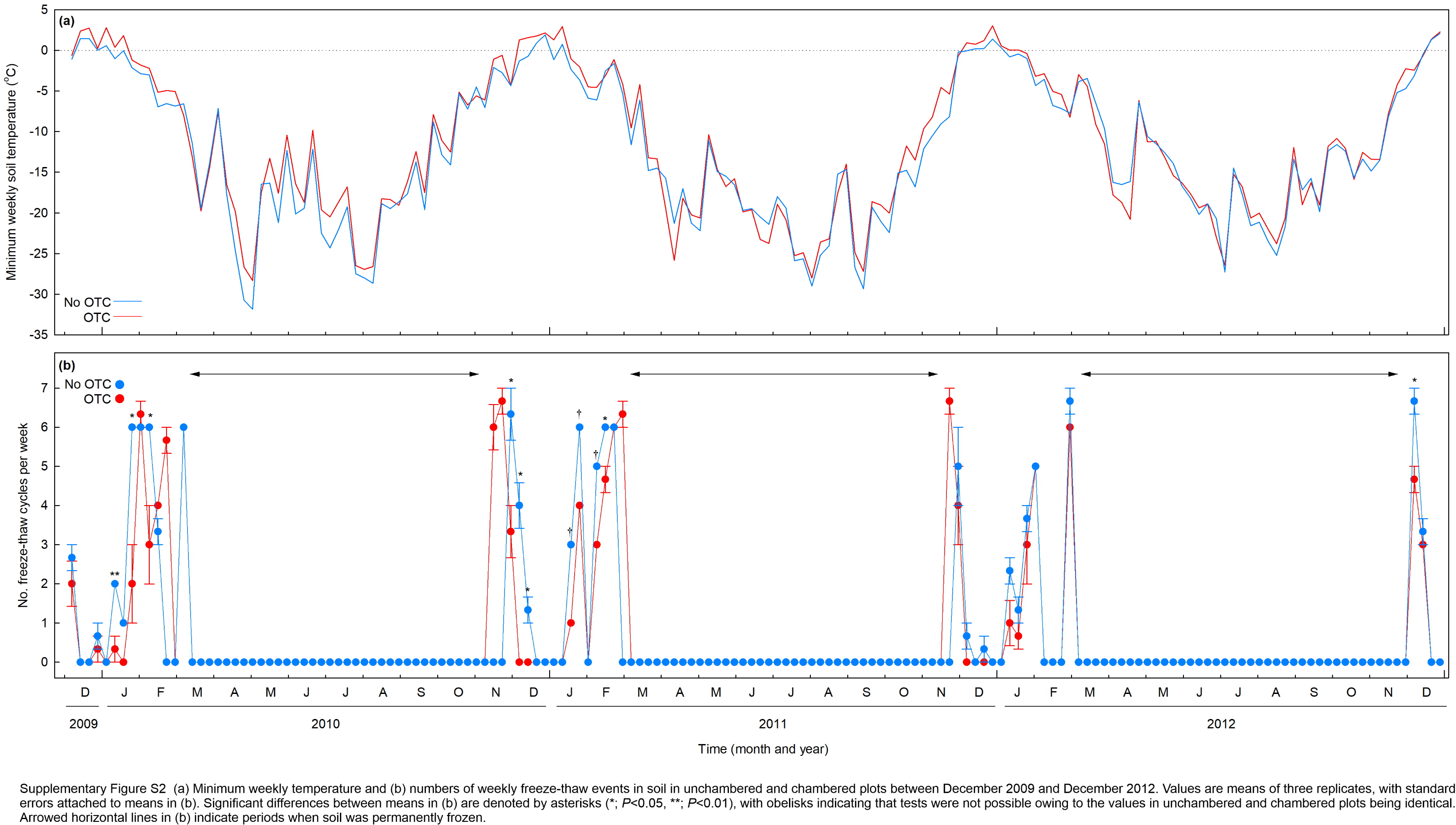
**

**
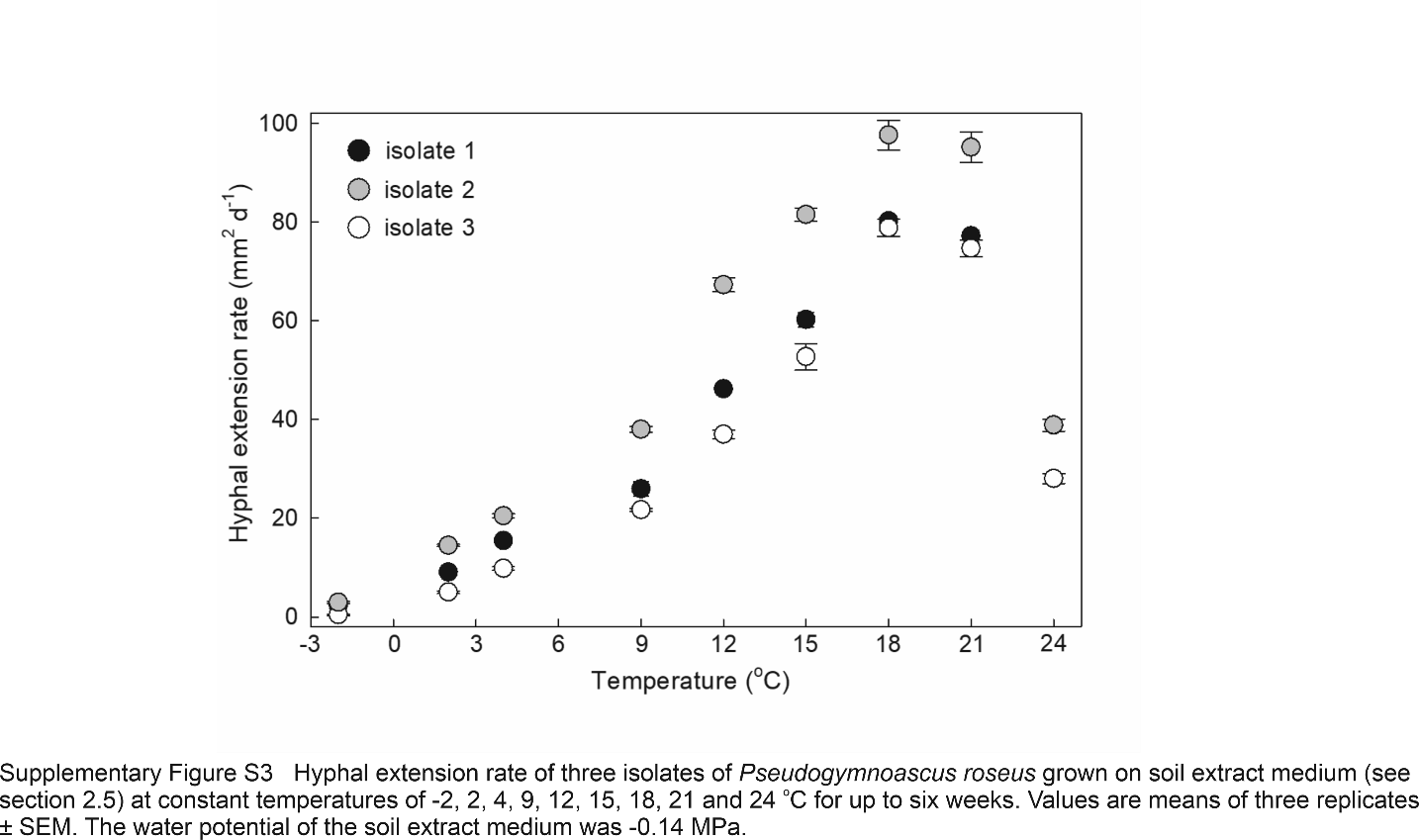
**


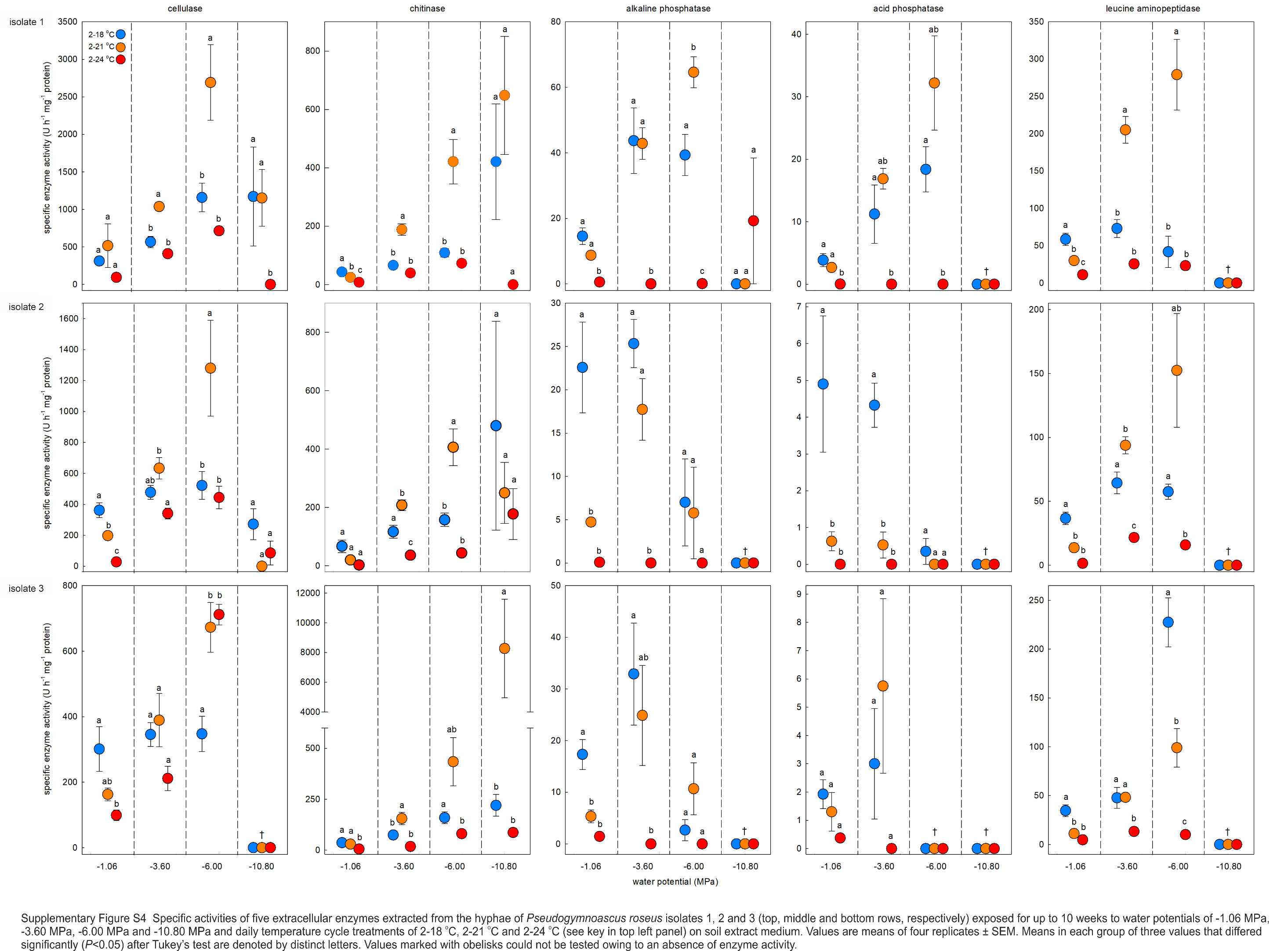

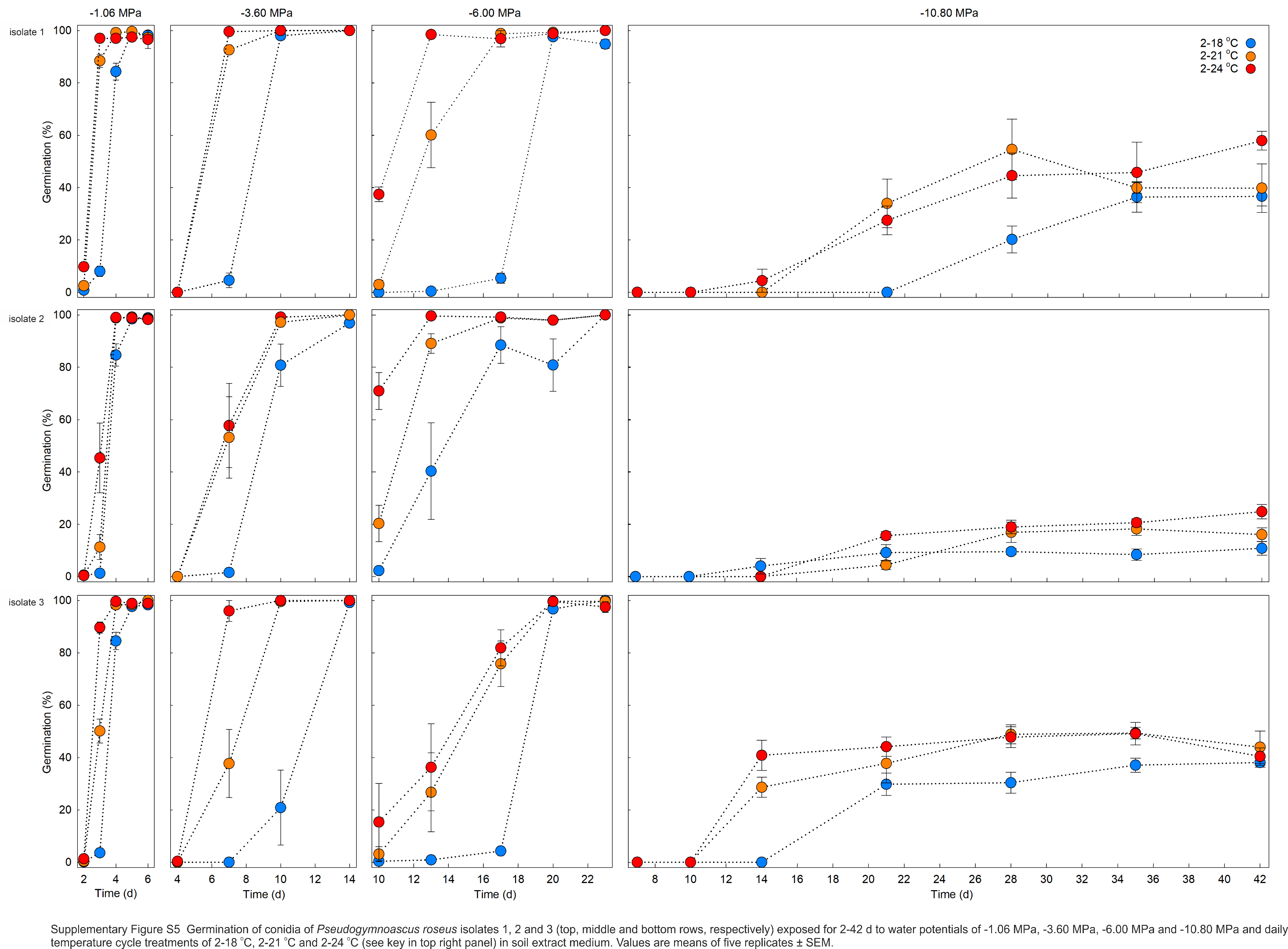


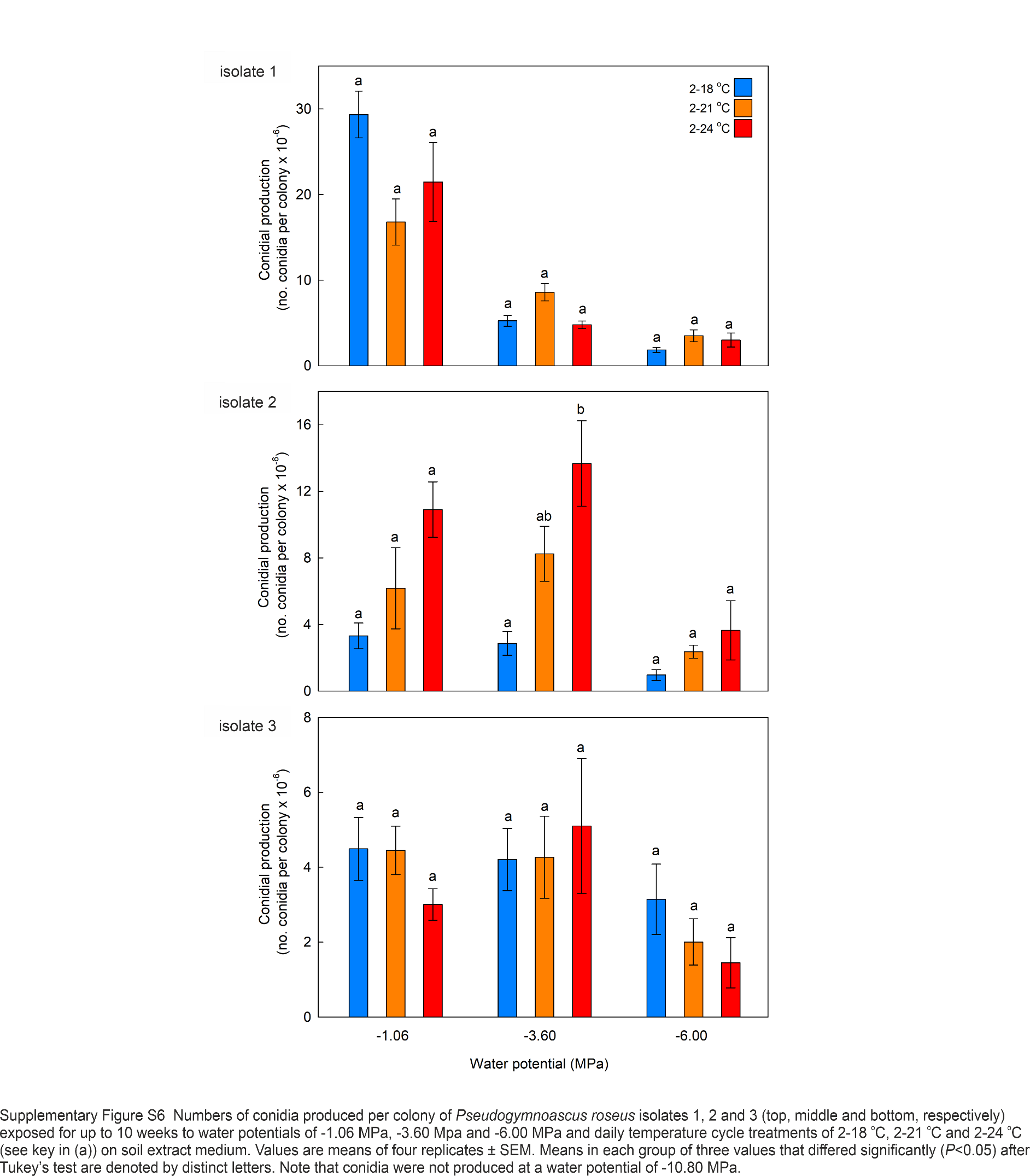

Supplement: Supplementary file 1 — Fig S1‐S6 [file GCB-27-1111-s001.docx]
